# Rate and predictors for non-attendance of patients undergoing hospital outpatient treatment for chronic diseases: a register-based cohort study

**DOI:** 10.1186/s12913-019-4208-9

**Published:** 2019-06-14

**Authors:** Donna Lykke Wolff, Frans Boch Waldorff, Christian von Plessen, Christian Backer Mogensen, Thomas Lund Sørensen, Kim Christian Houlind, Søren Bie Bogh, Katrine Hass Rubin

**Affiliations:** 1 Hospital of Southern Denmark, DK-6200 Aabenraa, Denmark; 2 Department of Regional Health Research, University of Southern Denmark, Winsløwparken 19, DK-5000 Odense C, Denmark; 3 Research Unit of General Practice, Department of Public Health, University of Southern Denmark, Odense, Denmark; 4 Direction Général de la Santé and Unisanté, Lausanne, Switzerland; 5 Department of Clinical Research, University of Southern Denmark, Odense, Denmark; 6 The Danish Patient Safety Authority, Kolding, Denmark; 7 Department of Vascular Surgery, Kolding Hospital, Part of Hospital Lillebaelt, Kolding, Denmark; 8 OPEN—Open Patient data Explorative Network— Department of Clinical Research and Odense University Hospital, Region of Southern Denmark, Odense, Denmark

**Keywords:** Non-attendance, No-show, Attendance rate, Chronic patients, Hospital outpatient clinic, Appointments, Predictors

## Abstract

**Background:**

Failure to keep medical appointments results in inefficiencies and, potentially, in poor outcomes for patients. The aim of this study is to describe non-attendance rate and to investigate predictors of non-attendance among patients receiving hospital outpatient treatment for chronic diseases.

**Methods:**

We conducted a historic, register-based cohort study using data from a regional hospital and included patients aged 18 years or over who were registered in ongoing outpatient treatment courses for seven selected chronic diseases on July 1, 2013. A total of 5895 patients were included and information about their appointments was extracted from the period between July 1, 2013 and June 30, 2015. The outcome measure was occurrence of non-attendance. The associations between non-attendance and covariates (age, gender, marital status, education level, occupational status, specific chronic disease and number of outpatient treatment courses) were investigated using multivariate logistic regression models, including mixed effect.

**Results:**

During the two-year period, 35% of all patients (2057 of 5895 patients) had one or more occurrences of non-attendance and 5% of all appointments (4393 of 82,989 appointments) resulted in non-attendance. Significant predictors for non-attendance were younger age (OR 4.17 for 18 ≤ 29 years as opposed to 80+ years), male gender (OR 1.35), unmarried status (OR 1.39), low educational level (OR 1.18) and receipt of long-term welfare payments (OR 1.48). Neither specific diseases nor number of treatment courses were associated with a higher non-attendance rate.

**Conclusions:**

Patients undergoing hospital outpatient treatments for chronic diseases had a non-attendance rate of 5%. We found several predictors for non-attendance but undergoing treatment for several chronic diseases simultaneously was not a predictor. To reduce non-attendance, initiatives could target the groups at risk.

**Trial registration:**

This study was approved by the Danish Data Protection Agency (Project ID 18/35695).

**Electronic supplementary material:**

The online version of this article (10.1186/s12913-019-4208-9) contains supplementary material, which is available to authorized users.

## Background

Patients who do not attend scheduled appointments in hospital outpatient clinics are a challenge for the health care system. Non-attendance is resource demanding, as missed appointments remain unused and new bookings must be made. Studies have associated non-attendance with an increased risk for hospitalizations and emergency department visits [1, 2]. Further, non-attendance may interrupt continuity of care resulting in reduced health outcomes e.g. impaired diabetes [1, 3, 4] and hyperlipidemia management [1].

A recent systematic review including studies in primary care and specialty clinics showed an average non-attendance rate of 23% with large variations according to medical specialties and continents [5]. Studies in Denmark have shown non-attendance rates of up to 14% [6, 7].

Research has often focused on non-attendance rates from the perspective of clinics [6, 8–12]; this study has chosen to focus on patients with chronic diseases. In the US and Europe, studies have reported that more than 40% of the population suffer from a chronic disease and approximately 20% live with multiple chronic diseases [13–17]. The prevalence of people with chronic diseases is expected to increase in the future as a result of aging populations, changed lifestyle and advances in healthcare [16, 18]. Patients with chronic diseases are usually in contact with the health care system for many years and the management of chronic disease accounts for approximately 80% of the health care budgets of Europe and the US [15, 17, 19]. Patients with multiple chronic diseases comprise up to 71% of the total health care budget [15, 17].

As economic resources are limited and workforces in the health care sector are under pressure from the rising demands posed by the increasing prevalence of patients with chronic diseases, there is an urgent need to manage chronic diseases more efficiently [20, 21]. One area of interest in this pursuit could be the reduction of resources spent on non-attendance [22].

In Denmark, the majority of patients with chronic diseases are managed by general practitioners, while patients with more complex diseases are referred for specialist treatment to hospital outpatient clinics [23, 24]. Knowledge about the prevalence of non-attendance and an understanding of its predictors may help to target interventions for optimizing attendance rates in this group of patients in need of long-term health care.

Many predictors of non-attendance have been examined as shown in a recent systematic review [5] but studies have often reported conflicting results as a consequence of the influences of patients, providers, and cultural contexts. In general, non-attendance may more often be predicted in those who are male, are young, are of a lower socioeconomic status, live a long distance from the clinic, have a prior history of non-attendance and have a long interval between the time at which their appointment was scheduled to the actual appointment date [5]. We find that there is a lack of studies on the predictive value of having specific diseases or several diseases. We are only aware of one such study, which found that patients with cardiac conditions have a lower non-attendance rate than those with other diseases [25].

Therefore, the aim of this study is to investigate non-attendance by patients undergoing hospital outpatient treatment for chronic disease. More specifically, the current project addresses the following research questions: First, what is the non-attendance rate of patients undergoing hospital outpatient treatment for chronic disease? Second, what are the predictors for non-attendance among this group, especially if undergoing treatment for several concurrent chronic diseases is a predictor for non-attendance?

## Methods

### Study population and setting

This study is a historic register-based cohort study. We included patients aged 18 years or older who were undergoing outpatient treatment for one or more selected common chronic diseases.

The common chronic diseases were chosen according to those defined as major by the Danish Health Authority and frequently encountered in hospital outpatient settings [26–28].

We included patients with type 1 diabetes mellitus (corresponding ICD–10 code: E10), type 2 diabetes mellitus (E11), heart failure (I50), other chronic obstructive pulmonary disease (COPD) (J44), asthma (J45), rheumatoid arthritis (M05), and osteoporosis (M80 and M81). Patients had to be in ongoing treatment on July 1, 2013 at Hospital Lillebaelt (Denmark).

Hospital Lillebaelt is a regional hospital with a rural and urban catchment population of nearly 300,000 inhabitants [29]. Information on patient appointments was extracted for the period from July 1, 2013 to June 30, 2015.

### Data sources

The Danish National Health Service provides access to tax-financed health care for Denmark’s 5.8 million citizens [30]. All Danish hospitals, public and private, report inpatient and outpatient contacts to the National Patient Register (NPR), which holds longitudinal data derived from the electronic medical records of all inpatients of Danish hospitals since 1977 and outpatients since 1994. NPR contains discharge diagnosis codes according to ICD-8 from 1977 to 1993 and adhering to ICD-10 from 1994 onwards [31]. Since 2000, it has also served as the basis for reimbursement via the Diagnostic Related Group system [32]. NPR comprises information on appointments that are actualized in attendance. However, NPR does not hold information on non-attendance as hospitals are not reimbursed for non-attended appointments. NPR was used to extract the study population and to obtain information on the patients’ age and gender as well as on all outpatient appointments during the study period.

As non-attendance is not registered in the NPR, we used information from Hospital Lillebaelt, where non-attendance is documented in the electronic medical records. Occurrences of non-attendance are registered on a daily basis, as health providers construct a schedule based on whether patients have attended, canceled, rebooked, or have not attended appointments. Non-attendance is not registered for the first appointment of the hospital outpatient treatment course.

Further, we gathered information on patients’ marital status from the Danish Civil Registration System [33], on their educational levels from the Danish Education registers [34], and on their occupational status and income from Danish registers on personal income and transfer payments at Statistics Denmark [35]. The links between data from different data sources are made possible using the unique 10-digit identification number assigned to all Danish citizens at birth or at first immigration [33].

### Outcome

The outcome variable was non-attendance. One appointment with or without attendance was included daily. If patients had two appointments on the same day with one registered as attendance and one registered as non-attendance, we categorized this day as attendance, as the non-attendance may have been caused by the hospital; for example, by a prolonged visit in one clinic or by a reservation of for medical imaging that was not used.

### Explanatory variables

Explanatory variables were chosen based on their influence on non-attendance according to previous studies [5]. Further, we included the variables “specific chronic disease” and “number of outpatient treatment courses” as we wanted to know the influence of disease and treatment characteristics. Information on the following explanatory variables was extracted:

- **Age** (categorized into 10-year intervals)**.** We used the patient age on July 1, 2013.
- **Gender** (categorized as male/female).
- **Marital status** (categorized as unmarried/married). The term “married” was used when patients were married or in a registered relationship on July 1, 2013. If no information existed on patients on the date of interest, we used their latest status.
- **Educational level** (categorized as basic (primary school)/vocational or upper secondary /further or higher education/unknown). For each patient, the highest completed level of education in 2013 was selected.
- **Occupational status** (categorized as student/ affiliated with the labor marked/short-term welfare payment/long-term welfare payment/disability pension/pension/unknown). Here, “short-term welfare payment” comprises individuals who were temporarily on a social income; for example, because of illness or maternity leave. “Long-term welfare payment” connotes individuals who received a social income for a longer period. “Disability pension” refers to individuals with a permanently reduced working capacity and a permanent social income. “Pension” describes individuals both on a pension and on an early pension. In our cohort, the retirement age was 65 years. The early pension (a self-paid settlement) can be received up to 5 years prior to the official retirement age. Information on this variable is reported on yearly basis and we extracted our data in 2013.
- **Specific chronic disease** (categorized as type 1 diabetes mellitus/type 2 diabetes mellitus/heart failure/COPD/asthma/rheumatoid arthritis/osteoporosis). Patients were categorized into one or more of the disease categories based on information about which outpatient treatment courses they were undertaking at Hospital Lillebaelt on July 1, 2013.
- **Number of outpatient treatment courses** (categorized as 1/2+). This was based on the number of specific hospital outpatient treated chronic diseases (out of the seven selected diseases) that each patient was undergoing on July 1, 2013.

### Statistical analyses

#### Analyses on the patient level

Patients were categorized as “attenders” if they attended all appointments during the 2 years and “non-attenders” if they missed one or more appointments. We described the patient characteristics of attenders and non-attenders using numbers and percentages and assessed their differences using chi-square tests.

#### Analyses on the appointment level

We reported non-attendance as a rate in proportion to appointments. We described patient characteristics both for appointments with attendance and for appointments with non-attendance. The outcome was reported in numbers and percentages and the differences were assessed using chi-square tests.

Associations between non-attendance and covariates were investigated using univariate and multivariate logistic regression models, including mixed effects to account for patients with multiple appointments. In the first multivariate logistic regression, we adjusted for age, gender, marital status, education level, occupational status, and specific chronic diseases. In the second multivariate logistic regression, we adjusted for the same variables except for specific chronic disease and we added the number of outpatient treatment courses. We used the Akaike information criterion (AIC) to choose the best model for our primary outcome regression.

All analyses were performed using Stata version 15 (StataCorp, College Station, TX, USA) and *p*-values of less than 0.05 were regarded as statistically significant.

### Ethics

The study did not involve any direct contact with patients or access to patient files. Thus, informed consent or ethical approval were not required [36]. The study was registered with the Danish Data Protection Agency (J.nr. 18/35695).

## Results

**Fig. 1:**
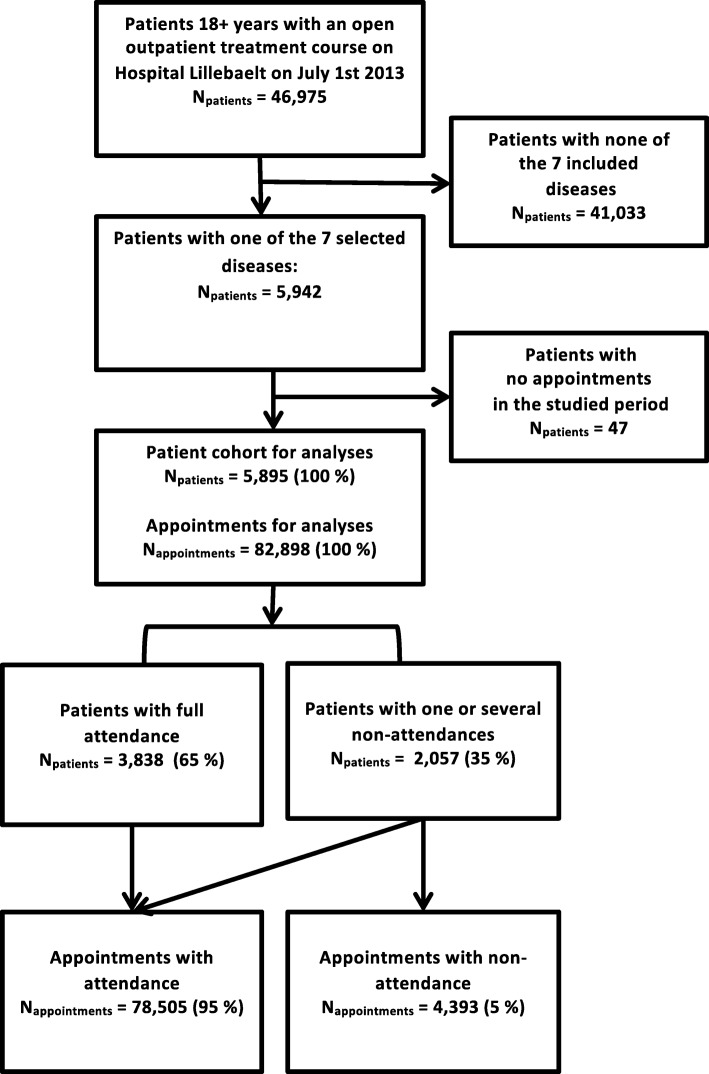
Flowchart of patient inclusion

On July 1, 2013, 46,975 patients were in hospital outpatient treatment at Hospital Lillebaelt (see Fig. 1: Flowchart of patient inclusion). A total of 5942 of these patients had one or several of the diseases selected for this study. We excluded 47 patients because they did not have any appointments during the study period, leaving 5895 available for analysis.

During the study period, 363 patients died. The mean follow-up time was 1.9 years.

Of the 5895 patients, 65% (3838 patients) attended all their appointments and 35% (2057 patients) missed one or several appointments in the two-year study period. The characteristics of patients with full attendance and patients with one or several occurrences of non-attendance are shown in Additional file 1.

### Results at the appointment level

The included patients had a total of 82,898 appointments during the study period, of which 5% (4393 appointments) were not attended (see Fig. 1: Flowchart of patient inclusion). A total of 195 appointments had occurrences of attendance and non-attendance on the same day; these were categorized as attendances.

The characteristics of patients who attended appointments and those who did not attend are shown in Table 1. There were significant differences in nearly all covariates between attendees and non-attendees. Those who did not attend appointments were more often young patients (40% vs. 19% aged 18–49 *p* < 0.001), male (58% vs. 52% *p* < 0.001), unmarried (53% vs. 42% *p* < 0.001) and low educated (44% vs. 41% with only primary school education *p* < 0.001). Retired patients had the lowest non-attendance rate of the occupational groups (31% vs. 53%). Among the specific disease groups, patients with type 1 diabetes (28% vs. 20% *p* < 0.001) had a higher non-attendance rate while a lower non-attendance rate was seen among patients with rheumatoid arthritis (11% vs. 14% *p* < 0.001) and osteoporosis (4% vs. 8% *p* < 0.001).

**Table 1:** Characteristics of appointments (*n* = 82,898 appointments)

Variable	Total		Appointments with attendance		Appointments with non-attendance		*p-value* ^*1*^
	n	%	n	%	n	%	
Overall	82,898	100%	78,505	100%	4393	100%	
Age							< 0.001
18–29	2329	3%	1925	2%	404	9%	
30–39	4682	6%	4202	5%	480	11%	
40–49	10,629	13%	9737	12%	892	20%	
50–59	15,989	19%	15,072	19%	917	21%	
60–69	25,364	31%	24,447	31%	917	21%	
70–79	18,659	23%	18,055	23%	604	14%	
80+	5246	6%	5067	6%	179	4%	
Gender							< 0.001
male	43,368	52%	40,830	52%	2538	58%	
female	39,530	48%	37,675	48%	1855	42%	
Marital status							< 0.001
Unmarried	35,678	43%	33,337	42%	2341	53%	
Married	47,220	57%	45,168	58%	2052	47%	
Educational level							< 0.001
Basic (primary school)	34,338	41%	32,413	41%	1925	44%	
Vocational or upper secondary	33,276	40%	31,601	40%	1675	38%	
Further or higher education	13,216	16%	12,563	16%	653	15%	
Unknown	2068	2%	1928	2%	140	3%	
Occupational status							< 0.001
Student	706	1%	599	1%	107	2%	
Affiliated to labour marked	21,185	26%	19,697	25%	1488	34%	
Short-term welfare payment	2028	2%	1844	2%	184	4%	
Long-term welfare payment	2996	4%	2646	3%	350	8%	
Disability pension	12,435	15%	11,649	15%	786	18%	
Pension	42,742	52%	41,359	53%	1383	31%	
Unknown	806	1%	711	1%	95	2%	
Specific chronic disease							
Type 1 diabetes mellitus							< 0.001
yes	17,310	21%	16,093	20%	1217	28%	
no	65,588	79%	62,412	80%	3176	72%	
Type 2 diabetes mellitus							0.53
yes	36,629	44%	34,762	44%	1867	42%	
no	46,269	56%	43,743	56%	2526	58%	
Hearth failure							0.15
yes	4129	5%	3928	5%	201	5%	
no	78,769	95%	74,577	95%	4192	95%	
COPD							0.53
yes	4854	6%	4582	6%	272	6%	
no	78,044	94%	73,923	94%	4121	94%	
Asthma							0.13
yes	5062	6%	4746	6%	316	7%	
no	77,836	94%	73,759	94%	4077	93%	
Rheumatorid arthritis							< 0.001
yes	11,621	14%	11,154	14%	467	11%	
no	71,277	86%	67,351	86%	3926	89%	
Osteoporosis							< 0.001
yes	6496	8%	6300	8%	196	4%	
no	76,402	92%	72,205	92%	4197	96%	
Number of outpatient treatment courses							0.32
1	79,719	96%	75,468	96%	4251	97%	
2+	3179	4%	3037	4%	142	3%	

^1^Univariate mixed effects logistic regression model for ‘appointments with attendance’ versus ‘appointment with non-attendance’ with a random effect taking appointments of the same paitent into account

The results of the multivariate logistic regression are shown in Table 2. In our primary multivariate logistic model (a) young patients (aged 18 ≤ 29 years) had the highest odds of missing their appointments (odds ratio (OR) 4.17; 95% confidence interval (CI) 2.70–6.42). Other significant predictors included middle-younger age (OR 2.58; CI 1.76–3.78 for 30 ≤ 39 years; OR 2.09; CI 1.47–2.98 for 40 ≤ 49 years), the male gender (OR 1.35; CI 1.20–1.52), being unmarried (OR 1.39; CI 1.24–1.56), having a low educational level (OR 1.18; CI 1.01–1.40), and being on long-term welfare payments (OR 1.48; CI 1.13–1.93). There were no specific diseases associated with higher odds of missing appointments.

**Table 2:** Appointment characteristic associated with non-attendences. Results of univariate and multivariate analysis for all variables: Odds ratios and their 95% conficence interval (*n* = 82,898 appointments)

	Univariate analysis		Multivariate analysis^a^		Multivariate analysis^b^	
	OR	(95% CI)	OR	(95% CI)	OR	(95% CI)
Age						
18–29	5.97*	4.28–8.33	4.17*	2.70–6.42	4.01*	2.63–6.11
30–39	3.25*	2.41–4.39	2.58*	1.76–3.78	2.51*	1.73–3.66
40–49	2.69*	2.06–3.53	2.09*	1.47–2.98	2.06*	1.45–2.93
50–59	1.53*	1.18–1.99	1.20	0.85–1.69	1.18	0.84–1.67
60–69	0.87	0.68–1.13	0.88	0.68–1.15	0.88	0.67–1.15
70–79	0.85	0.65–1.11	0.90	0.69–1.18	0.90	0.69–1.18
80+	1		1		1	
Gender						
Male	1.36*	1.21–1.53	1.35*	1.20–1.52	1.44*	1.29–1.61
Female	1		1		1	
Marital status						
Unmarried	1.67*	1.49–1.88	1.39*	1.24–1.56	1.40*	1.25–1.57
Married	1		1		1	
Educational level						
Basic (primary school)	1.14	0.96–1.35	1.18*	1.01–1.40	1.21*	1.03–1.43
Vocational or upper secondary	1.00	0.85–1.18	0.95	0.81–1.12	0.96	0.82–1.13
Further or higher education	1		1		1	
Unknown	1.67*	1.16–2.42	1.71*	1.21–2.42	1.77*	1.25–2.50
Occupational status						
Student	2.24*	1.40–3.59	0.83	0.50–1.38	0.83	0.50–1.38
Affiliated to labor marked	1		1		1	
Short-term welfare payment	1.59*	1.13–2.23	1.32	0.95–1.83	1.35	0.97–1.89
Long-term welfare payment	1.88*	1.44–2.45	1.48*	1.13–1.93	1.50*	1.15–1.95
Disability pension	0.96	0.80–1.14	1.08	0.90–1.30	1.12	0.93–1.34
Pension	0.43*	0.38–0.49	0.75*	0.59–0.96	0.76*	0.59–0.97
Unknown	2.13	1.31–3.46	1.99*	1.24–3.19	2.05*	1.27–3.30
Specific diseases						
Type 1 diabetes mellitus						
yes	1.75*	1.53–2.01	1.04	0.73–1.48		
no	1		1			
Type 2 diabetes mellitus						
yes	0.96	0,86-1,08	1.14	0.81–1.59		
no	1		1			
Hearth failure						
yes	0,81	0,61-1,08	1.16	0.78–1.70		
no	1		1			
COPD						
yes	1,08	0,85-1,36	1.44	0.99–2.10		
no	1		1			
Asthma						
yes	1,19	0,95-1,49	0.96	0.66–1.41		
no	1		1			
Rheumatorid arthritis						
yes	0,71*	0,60-0,85	0.95	0.67–1.34		
no	1		1			
Osteoporosis						
yes	0,43*	0,34-0,54	0.79	0.54–1.15		
no	1		1			
Number of outpatient treatment courses						
1	1				1	
2+	0,84	0,59-1,19			1.05	0.76–1.46

^a^adjusted for age, gender, marital status, education level, occupational status and specific chronic disease. AIC = 30,456

^b^adjusted for age, gender, marital status, education level, occupational status and number of outpatient treatment courses. AIC = 30,464

*p < 0.05

The secondary multivariate logistic regression (b) showed that patients undertaking several outpatient treatment courses were not significantly different from patients undertaking one outpatient treatment course.

## Discussion

This study of patients undergoing routine hospital outpatient treatments for chronic diseases found that 35% of patients had one or more occurrences of non-attendance and 5% of all appointments were not attended. Youth, the male gender, an unmarried status, low educational levels, and receipt of long-term welfare payments were all predictors of non-attendance. We did not find, that undergoing treatment for several chronic diseases simultaneously was a predictor for a higher rate of non-attendance.

We found a 5% rate of non-attendance, a lower rate than the average 23% reported in a newly published systematic review on non-attendance [5]. However, non-attendance rates show large differences across specialties as well as continents. For example, the median rate of non-attendance found by studies in cardiology was 30%, while the median rate was 36% in endocrinology. However, researchers report that some studies define the non-attendance rate as including rescheduled or canceled appointments [5]. This naturally results in a higher rate than when non-attendance is defined only as appointments that have been missed, as we have used in our study. Our results are more consistent with Danish studies. Studies conducted in 2003 on several different Danish hospital outpatient clinics showed variations in non-attendance from 0 to 14%, in average between 3 and 4% [7]. A recent Danish study on radiology and orthopedic clinics likewise showed 4 and 5% non-attendance rates, which were similar to our findings [6].

We think that the relatively low rate of non-attendance that we found, in contrast to international studies but concurrent with Danish studies, might be explained by factors such as age, geographical distance, and an aspect of culture. Although we found that young age was associated with higher non-attendance rates, confirming the results of other studies [5], the majority of patients in our study were elderly (77% were 50 years or older) and, therefore, the overall non-attendance rate was lowered. Additionally, distance from the hospital has been associated with higher non-attendance rates [5], but the majority of patients in Denmark do not reside a long distance from the nearest hospital; for instance, in the hospital in our study the maximum travel time was 1 h by car. Further, in Denmark we have a good public transport system. The Danish study on radiology and orthopedic clinics included travel time in their investigation and found no association with higher non-attendance rates [6].

We suspect there may be a cultural aspect to these findings as well, in that Danes, for the most part, either attend or give notice when they have an appointment. We find it notable that Danish patients are not charged a fee for non-attendance, and at the time this study was conducted, neither did they receive a reminder about appointments prior to the day of attendance.

We found several significant predictors for non-attendance. Our findings are concurrent with the systematic review by Dantas and colleagues that showed that young age and low socioeconomic status are predictors for non-attendances [5]. With regard to gender and marital status the review showed that the majority of studies did not find any statistical differences between these factors in relation to non-attendance. However, the few studies that did find a difference, are concurrent with our findings, that male gender and unmarried status are predictive factors for a higher non-attendance rate.

The novel finding of this study – that patients undertaking several outpatient treatment courses do not have higher rates of non-attendance – is surprising as we hypothesized that patients with several hospital outpatient treated diseases may have several appointments to keep in different clinics, and therefore, would require a higher degree of coordination, which could cause a greater rate of non-attendance. Our finding contradicts this, and we reflect that patients treated for long-term chronic diseases may be used to the routine of attendance. It can be the on-going relation with the health care team that nurses them to come or maybe their diseases are more severe and medical assistance such as medicine adjustments are required more often and motivates this patient group to show up.

### Strengths and limitations

Our study has important strengths. First, it included all patients undergoing hospital outpatient treatment for the selected diseases at Hospital Lillebaelt. Second – information from other registries for all patients was available because of the unique identification number. Third, the diagnoses in NPR are generally of high validity [31]. Of the diagnoses we included, only osteoporosis has not been validated in a hospital outpatient setting. The positive predictive value (PPV) for each of the other diagnoses is as follows: type 1 diabetes and type 2 diabetes (PPV = 96.0 (86.5–98.9)), heart failure (PPV = 100 (92.9–100)), COPD (PPV = 100 (92.9–100)), asthma (PPV = 65.3 (62.2–68.3)), and rheumatoid arthritis (PPV = 98.0 (89.5–100)) [31]. Third, we had a large study sample including patients from different hospital clinics.

However, there are also some limitations to our study. First, it is based on administrative data. As all attended appointments are financially reimbursed by the government, we presume that the data are complete. In contrast, appointments without attendance are not financially reimbursed, and although there is a policy at Hospital Lillebaelt of documenting non-attendance, we cannot be sure, that it is always registered correctly. A limitation is also the possibility of non-attendance being caused by hospital admission, though admissions are often known in the documenting process of non-attendance. Further, the database of Lillebaelt does not record missed appointments that would have provided the first contact for a new outpatient treatment course. This possibly introduced a component of measurement bias to the study, resulting in the under- or overestimation of the reported rate. However, the predictors would presumably have remained unaffected, as the bias would have been equally distributed within the groups of attendees and non-attendees, thereby retaining their proportions. Additionally, we chose to categorize information on patients who attended an appointment and missed an appointment on the same day under attended appointments. As this was the case for only 195 appointments, it could not have biased the results. Nonetheless, a limitation can be found in that the collection of information on predictors was extracted on July 1, 2013, thereby earlier than the actual appointments. This may have resulted in an under- or overestimation of the “number of outpatient treatment courses.” We presume that variations in the other predictors have not greatly affected the overall results as they are unlikely to have varied significantly over the two-year study period.

While we set out to investigate the effect of having multiple chronic diseases on the rate of non-attendance, we only investigated diseases treatment at hospital outpatient level. Patients may have other chronic diseases that they undergo treatment for in primary care. Therefore, we can only investigate the effect of having multiple chronic diseases at a hospital outpatient level.

In this study we investigated factors we could extract from the administrative system which do not contain information on patient reported outcome measures. Information regarding the patient’s perception could be investigated by means of qualitative interviews. We did not investigate if visits were in the form of face-to-face meeting, telephone consultations or other kind of telehealth communication which could have been relevant for subgroup analysis. Furthermore, it was not possible to divide appointments based on their purpose, e.g., acute appointment; evaluate a medication dosage change; routine follow-up to monitor chronic conditions; etc. which is a limitation.

Finally, hospital Lillebaelt has a rural and urban catchment population that covers 5% of the Danish population, but other Danish hospitals may differ in catchment population. However, overall we find our conclusions applicable to other Danish hospitals and health-care systems like the Danish.

### Clinical implications

This study showed that Danish patients with chronic diseases miss one in every 20 appointments (5%). A major part of hospital outpatient activity involves patients in treatment for chronic diseases and non-attendance by members of this group is very inefficient for the hospital. Further, perhaps especially for patients with chronic conditions, continuity of care is an important aspect and non-attendance interrupts this continuity. Therefore, we argue that a further reduction in the non-attendance rate is important.

The results from our study on chronic patients in hospital outpatient clinics can be made operational in a number of ways. Knowledge about predictors for potential non-attenders can assist in targeted interventions; for example, studies on outpatient reminder systems have shown that they reduce non-attendance [12, 37]. In a hospital were such a system has been implemented it is relevant to investigate to what extent this affects non-attendance rates among chronic patient groups.

In this study we investigated the extent of non-attendance for patients in long-term treatment and some factors for non-attendance. Information regarding mechanisms on why these patients do not attend their appointments by qualitative methods should be obtained e.g., if non-attendance is caused by forgetfulness a reminder system could help, but patients may miss appointments for other reasons that are important to discover if we wish to find better ways to organize their treatment courses.

A final note on the clinical implication of this study regards the clinical relevance of the prognostic factors. We find our results robust because of the relatively large sample size. It is however for the interested clinic or hospital management to decide which prognostic factors are of clinical relevance which will depend on factors such as context and population size.

## Conclusion

In conclusion, our study showed that among the group of patients in hospital outpatient treatment for chronic disease one in every 20 appointments (5%) resulted in non-attendance. We found several predictors for non-attendance including youth, male gender, unmarried status, low educational level, and the receipt of long-term welfare payments. We found no specific patient groups associated with a higher non-attendance rate. Neither did patients treated for several chronic diseases have a higher rate of non-attendance than patients in hospital outpatient treatment for a single chronic disease. As continuity of care is important for patients with chronic diseases and non-attendance is inefficient for the health care system our findings can be used to assist in targeting groups for interventions to reduce the non-attendance rate. Nonetheless, further research on why these chronic patients miss appointments is required.

## Additional file

Additional file 1: “Characteristics of patients with full attendance (attenders) and partial attendance (non-attenders) during the two year study period (*n* = 5,895 patients)”. (PDF 45 kb)

## Data Availability

The data that support the findings of this study are available at Statistic Denmark, but restrictions apply to the availability of these data, which were used under license for the current study, and so are not publicly available. Data are however available from the authors upon reasonable request and with permission of the Danish Data Protection Agency and Statistic Denmark.

## Abbreviations

AIC: Akaike information criterion
COPD: Chronic obstructive pulmonary disease
