# Supplementary material for: Rate and predictors for non-attendance of patients undergoing hospital outpatient treatment for chronic diseases: a register-based cohort study
Source: BMC Health Serv Res. 2019 Jun 14;19:386. doi: 10.1186/s12913-019-4208-9 (PMC6570866; doi:10.1186/s12913-019-4208-9)
Supplement: Supplementary file 1 — “Characteristics of patients with full attendance (attenders) and partial attendance (non-attenders) during the two year study period (n = 5,895 patients)”. (PDF 45 kb) [file 12913_2019_4208_MOESM1_ESM.pdf]

Additional file 1: Characteristics of patients with full attendance (attenders) and partial attendance (non-attenders) during the two year study period (n = 5,895 patients)

Variable	Total		Attenders		Non-attenders		
	n	%	n	%	n	%	p-value[1]
**Overall**	5,895	100%	3,838	100%	2,057	100%	
**Age**							<0.001
18-29	206	3%	74	2%	132	6%	
30-39	383	6%	192	5%	191	9%	
40-49	778	13%	426	11%	352	17%	
50-59	1,183	20%	750	20%	433	21%	
60-69	1,700	29%	1,222	32%	478	23%	
70-79	1,251	21%	894	23%	357	17%	
80+	394	7%	280	7%	114	6%	
**Gender**							<0.001
male	2,959	50%	1,833	48%	1,126	55%	
female	2,936	50%	2,005	52%	931	45%	
**Marital status**							<0.001
Unmarried	2,519	43%	1,515	39%	1,004	49%	
Married	3,376	57%	2,323	61%	1,053	51%	
**Educational level**							0.003
Basic (primary school)	2,351	40%	1,500	39%	851	41%	
Vocational or upper secondary	2,345	40%	1,550	40%	795	39%	
Further or higher education	1,054	18%	711	19%	343	17%	
Unknown	145	2%	77	2%	68	3%	
**Occupational status**							<0.001
Student	63	1%	29	1%	34	2%	
Affiliated to labour marked	1,838	31%	1,138	30%	700	34%	
Short-term welfare payment	125	2%	61	2%	64	3%	
Long-term welfare payment	200	3%	88	2%	112	5%	
Disability pension	718	12%	411	11%	307	15%	
Pension	2,896	49%	2,086	54%	810	39%	
Unknown	55	1%	25	1%	30	1%	
**Specific chronic disease**							
Type 1 diabetes mellitus							<0.001
yes	1,144	19%	657	17%	487	24%	
no	4,751	81%	3,181	83%	1,570	76%	
Type 2 diabetes mellitus							0.01
yes	2,281	39%	1,439	37%	842	41%	
no	3,614	61%	2,399	63%	1,215	59%	

**Hearth failure**								0.09
	**yes**	287	5%	200	5%	87	4%	
	**no**	5,608	95%	3,638	95%	1,970	96%	
**COPD**								0.5
	**yes**	425	7%	283	7%	142	7%	
	**no**	5,470	93%	3,555	93%	1,915	93%	
**Asthma**								0.73
	**yes**	454	8%	299	8%	155	8%	
	**no**	5,441	92%	3,539	92%	1,902	92%	
**Rheumatorid arthritis**								0.18
	**yes**	752	13%	473	12%	279	14%	
	**no**	5,143	87%	3,365	88%	1,778	86%	
**Osteoporosis**								<0.001
	**yes**	688	12%	562	15%	126	6%	
	**no**	5,207	88%	3,276	85%	1,931	94%	
**Number of outpatient treatment courses**								0.02
**1**		5,760	98%	3,763	98%	1,997	97%	
**2+**		135	2%	75	2%	60	3%	

[1] Chi-square test between attenders and non-attenders
